# Bio-efficacy of deltamethrin based durable wall lining against wild populations of *Anopheles gambiae* s.l. in Northern Tanzania

**DOI:** 10.1186/s13104-017-2414-2

**Published:** 2017-02-10

**Authors:** Eliningaya J. Kweka, Ming-Chieh Lee, Beda J. Mwang’onde, Filemoni Tenu, Stephen Munga, Epiphania E. Kimaro, Yousif E. Himeidan

**Affiliations:** 1 Division of Livestock and Human Diseases Mosquitoes Control, Mosquito Section, Tropical Pesticides Research Institute, P.O. Box 30214, Arusha, Tanzania; 2 Department of Medical Parasitology and Entomology, Catholic University of Health and Allied Sciences, P.O. Box 1464, Mwanza, Tanzania; 3 Program in Public Health, College of Health Sciences, University of California Irvine, Irvine, CA USA; 4 Amani Medical Research Centre, National Institute for Medical Research, P.O. Box 81, Muheza, Tanzania; 5 Centre for Global Health Research, Kenya Medical Research Institute, P.O.Box 1578, Kisumu, Kenya; 6 Africa Technical Research Centre, Mosquitoes Health International, P.O. Box 15500, Arusha, Tanzania

**Keywords:** Durable wall lining, Indoor residual spray, Culicine, *Anopheles gambiae* s.l., Bioassays, Resistance, Susceptible

## Abstract

**Background:**

Indoor residual spraying (IRS) is one of the preferred tools used for control of malaria in many settings in the world. However, this control tool still faces challenges that include lack of long lasting active ingredient, limited number of well-trained personal, and need of repeated treatment which increases operational costs and reduces acceptability by residents. As a result there is need to develop and validate other methods which can complement the existing controls. The current study compared the bio-efficacy of durable wall lining (DL) (treated with deltamethrin 265 mg/m^2^) and IRS (with deltamethrin 5% WP at 20 mg/m^2^) on indoor mosquitoes densities and biting behaviour of mosquitoes in comparison with control houses without either DL or IRS.

**Methods:**

A study with two treatment arms and a control was conducted in Magugu ward, Northern Tanzania. Overall, a total of 60 houses were selected for the study with 20 houses per treatment arm and control. From each arm and control five houses were selected for mosquitoes trapping. Mosquitoes were sampled from 18:00 to 07:00 hourly every month for a period of 6 months. Mosquitoes were sampled using CDC miniature light traps.

**Results:**

A total of 14,400 female wild mosquitoes were used for contact bioassays in the control arm. 20 houses were sprayed, additionally walls of 20 houses were installed with wall liners, and walls of 20 unsprayed houses were used as control. Also, a total of 946 mosquitoes were sampled with traps in 60 houses during the hourly sampling for 6 months. A total of 3000 unfed females of *An. gambiae* s.l. wild population raised from larvae were collected from natural habitats in the same village for bioassays. The decline in indoor mosquitoes densities observed in this study did not lead to a shift in the biting cycles (*P* = 0.712). The number of mosquitoes caught indoors in houses with DL and IRS was significantly lower (*P* < 0.001) compared to control houses. When the comparisons were done between DL and IRS houses, the densities were significantly lower in DL houses compared to IRS houses (*P* = 0.021). In the DL installed houses, indoor mosquito density declined notably and sustained throughout the 6 months of the study. However, in those houses sprayed with deltamethrin 5% WP (PALI™5 WP), the density noted to start to increase within four months after spraying(do you mean to say that the densities declined up to 4 months post spraying and thereafter increased.

**Conclusions:**

Considering the efficacy duration of DL against IRS with deltamethrin 5% WP on mosquito densities decline indoors. The results of this study suggest that DL is more effective in malaria control as its efficacy lasted more than that of IRS.

## Background

Currently, use of long lasting insecticide treated nets (LLINs) and indoor residual spray (IRS) are the preferred tools for mosquitoes control in sub Saharan Africa [1–3]. The main mosquitoes observed in this region are *Anopheles gambiae* complex sibling species and *An. funestus* group [4–6]. It has been reported that the biting and host seeking behaviour of *An. gambiae* s.l. and *An. funestus* starts at dusk but the frequency increases from 22:00 h to mid-night. This biting cycle eventually peaks again in the morning starting at 3.00 to 6.00 a.m. [7, 8]. Recent studies have suggested early biting behaviour in *An. gambiae* s.l., starting from 19:00 and 20:00 h and this has been attributed to the increased distribution and coverage of LLINs [9].

In Tanzania there has been scaling up of conventionally treated nets and LLINs since 1980s and 2005 respectively [10–12]. In some malaria endemic foci, LLINs have been combined with IRS [2, 13] and incidences of malaria have significantly decreased in areas with increased coverage of LLINs use [3, 14]. More recently there has been concern of the impact of LLIN on mosquitoes as mosquitoes have been found resting indoor in houses treated with LLINs or IRS due to insecticides resistance selection pressure among mosquitoes population hence delayed knockdown on the surface of the net or walls [15, 16]. This suggests that in areas where the main mosquitoes is *An. arabiensis*, adding IRS into houses with LLINs does not enhance house-hold level protection except where the IRS employs non-pyrethroid insecticides [2, 17]. More recently durable wall linings (DL) have been produced and this study tested whether they have greater impact in reducing indoor resting mosquitoes compared to normal IRS. The same active ingredient (AI) of deltamethrin was used in both DL and IRS. The DL was incorporated with deltamethrin at a dosage of 265 mg/m^2^ [3]. The compound used for IRS was PALI™ 5 WP (Deltamethrin 5% WP) the dosage is 20 mg/m^2^ which was diluted in 10 l of water and applied in 250 m^2^.

The objective of current study was to compare the bio-efficacy of durable wall lining (DL) (treated with deltamethrin 265 mg/m^2^) and IRS (with deltamethrin 5% WP at 20 mg/m^2^) on mosquito indoor mosquitoes densities and biting behaviour in comparison with control houses without either DL or IRS.

## Methods

### Study sites

**Fig. 1:**
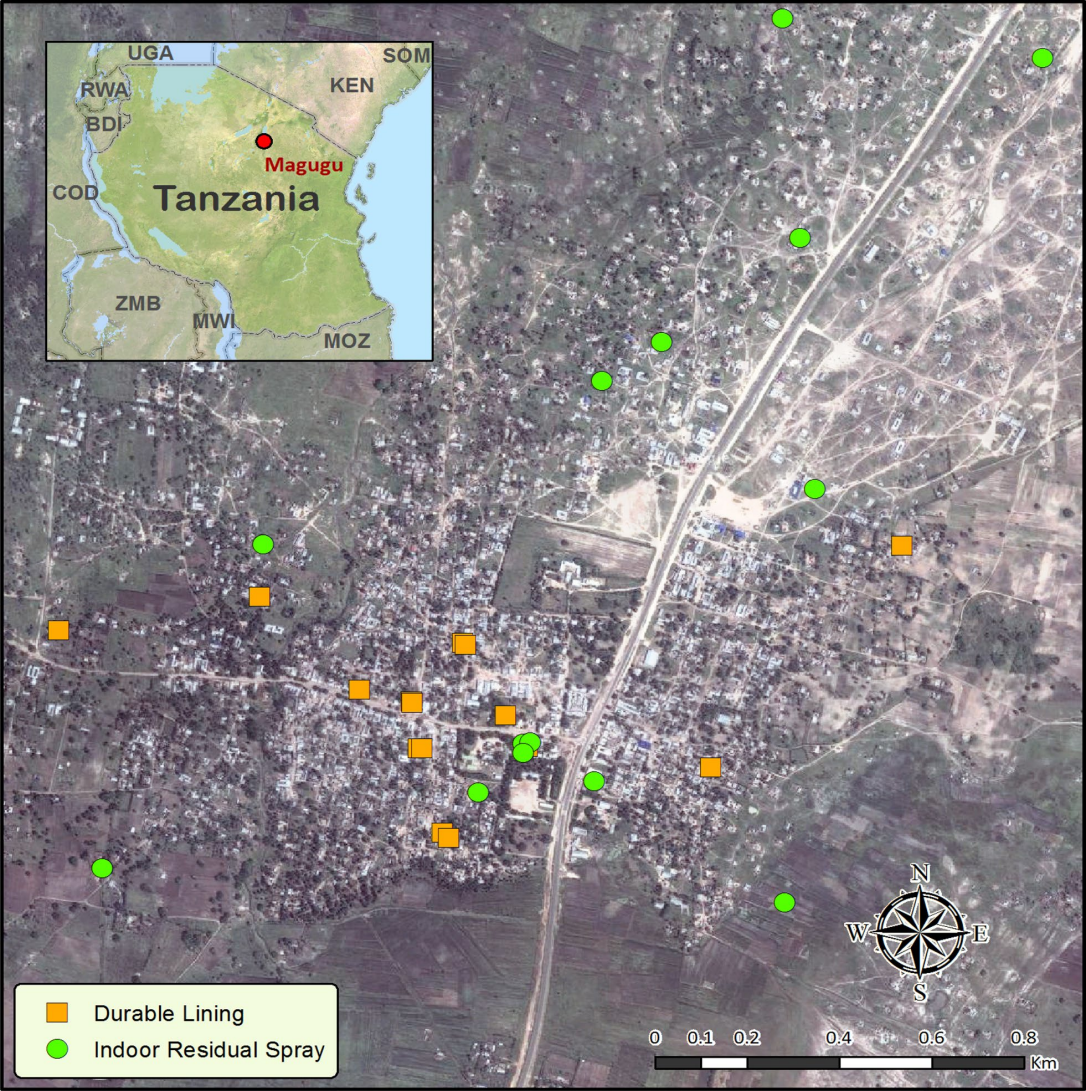
The map of Tanzania showing the study site in Great Northern Rift Valley of Tanzania (The GIS points are our original work and the background of this map is developed from Google map)

The study was conducted in, Magugu ward in Babati district (malaria epidemic prone site), Manyara region. This study site is located in the Great Rift Valley of northern Tanzania. Twenty houses were selected (give the basis for selecting the 20 houses—were they randomly selected) for DL installation and other twenty for IRS. Trial houses were labelled and mapped (Fig. 1). Houses were selected based on household agreement. The three arms (i.e. wall liner, IRS and control) were selected clustered together. Twenty houses each in three arms each were selected for follow up during the 6 month study period. Baseline sampling was conducted before treatment in December, 2012. The L1014F mutation in this study area was found in *An. arabiensis* at the allelic frequency of 11.5% in Babati by previous study [10, 18]. In this site, kdr mutations were recorded without obvious phenotypic resistance to pyrethroids being observed [10, 18].

### *Anopheles gambiae* s.l. larvae sampling

*Anopheles gambiae* s.l. larvae were sampled in breeding sites such as drainage ditches, abandoned brick pits, pottery and tyres obsoletes for six consecutive months. Mosquito larvae specimen were collected using a standard 350 ml dipper (BioQuip Products, Inc. California, USA) and transported to the laboratory and reared to adults in the insectary at the Tropical Pesticides Research Institute, Magugu field station and identified using morphological keys developed by Gillies and Coetzee [19]. The emerged adults were used for the susceptibility tests.

### Mosquito indoors sampling by traps

Twenty houses were selected for each treatment arm and control. Mosquitoes were sampled using CDC miniature light traps from 18:00 to 07:00 hourly monthly for 6 months [20, 21]. Sampling was done from 18:00 to 07:00 h at hourly interval to monitor the effect of the protective efficacy of control tools on biting cycle and house entry behaviour. The collected mosquitoes were kept in a well labelled paper cup, then transported to the laboratory for morphological species identification [19].

### Insecticide susceptibility tests

Insecticide susceptibility tests were conducted with wild population mosquitoes to ascertain the efficacy of the control tools against wild mosquito populations. Bioassay tests were carried out using the standard WHO protocol [22], using wild and laboratory colony mosquitoes. Insecticide susceptibility test kits and impregnated papers approved by WHOPES with discriminating dosage was used [23]. Larvae were reared in insectary from wild sampled population of *An. gambiae* s.l. and 2 day old non-blood-fed adult female of *An.* *gambiae* s.l. wild population used in the tests. Batches of 25 mosquitoes per replicate were exposed to test papers impregnated with bendiocarb (0.1%), DDT (4%), permethrin (0.75%) and deltamethrin (0.05%). Each test had four replicates and two controls. Deltamethrin is the active ingredient used in both IRS and DL. In the control experiments, wild population of mosquitoes from the same sites were exposed to untreated papers (Standard WHO control papers for each insecticides class). The knockdown effect of each insecticide was recorded in time interval 10, 15, 20, 30 40, 50, 60 min, and mortality recorded 24 h post test as scheduled in WHO protocol. Mosquitoes were then transferred to a paper cup and provided with 10% glucose solution. Final mortality was recorded 24 h post-exposure. WHOPES suggests that, if 98–100% mosquito mortality is observed, this indicate insecticide susceptibility, mortality <98% suggests existence of resistance that needs to be confirmed, and mortality <90% mortality suggests resistance. All batches of insecticide-impregnated paper (WHOPES approved standard papers) were pre-tested on a laboratory strain of *An. gambiae* s.s. maintained at the insectary, which is known to be highly susceptible to pyrethroids and DDT. All susceptibility tests were carried out at 26–29 °C and 74–82% relative humidity.

### Contact bioassays

The persistence of biological efficacy of the insecticide formulations on the sprayed surfaces of the houses and those covered by DL was determined by contact bioassays. Wild population of *An. gambiae* s.l. were exposed to the sprayed walls using standard cones [24]. Into each cone 10 blood-fed mosquitoes, obtained from daytime resting collections in human dwellings and cowsheds of the same village, were released and exposed for 30 min and then removed using an aspirator. Controls were exposed to an unsprayed surface. Each test batch of mosquitoes was held in a paper cup covered with netting and provided with a cotton pad of glucose solution (10% sugar solution). Knockdown and mortality was recorded after 1, and 24 h respectively at room temperature. When mortality in control exceeded 20%, results were rejected. Bioassay was done in day 1 and thereafter was done monthly for 6 months. The spray-man and head of household were interviewed on the spray day up to 6 months to know the perceived side effects.

### Data analysis

Data analysis was performed using the PWAS statistic version 18.0 (SPSS Inc., Chicago, IL). Due to low number of mosquitoes sampled hourly, the number of mosquitoes were log transformed [log (n + 1)]. One way Analysis of variance (ANOVA) was used to compare mosquitoes ‘abundance in the two treatment arms and a control. A paired sample *T* test, homoscedastic, was used to compare performances between DL and IRS. The proportions of mortality between IRS and DL walls were compared using Chi square test. The time lapse in months between treatment and mosquito population’s resurgence was analyzed using generalized linear model (GLM), univariate analysis density being a dependent variable.

## Results

### Mosquitoes sampling by CDC light traps

Overall, a total of 946 mosquitoes were collected from the DL, IRS treated and control houses over the 6 month period. Out of the total number sampled, 392 (41.4%) were Culicine species, 553(58.5%) *An. gambiae* s.l. and 1 (0.1%) *An. funestus* (Table 1). *An. funestus* was not included in the analysis because of low proportion vis-à-vis Culicines and *An. gambiae* s.l. There were no significant differences (*P* > 0.05) within treated houses in mosquito densities within each treatment arm therefore, all houses were pooled together in respective hours and treatment. The total of 14,400 female *An. gambiae* s.l. females were used for the contact bioassays in the walls of houses sprayed with deltamethrin, Walls installed with DL and control houses.

**Table 1:** Summary for the mosquito’s species sampled with CDC miniature light trap from 18:00 to 07:00 h before and after the interventions

Species	Treatment	December	January	February	March	April	May	June	Total
*Anopheles funestus*	DWL	1	0	0	0	0	0	0	*1*
*Anopheles funestus*	IRS	0	0	0	0	0	0	0	*0*
*Anopheles funestus*	Control	0	0	0	0	0	0	0	*0*
*Anopheles gambiae* s.l.	DWL	73	4	0	0	0	0	0	*77*
*Anopheles gambiae* s.l.	IRS	65	5	6	6	7	16	17	*122*
*Anopheles gambiae* s.l.	Control	80	35	49	49	42	48	51	*354*
*Culex species*	DWL	37	12	9	2	0	0	0	*60*
*Culex species*	IRS	38	7	16	0	9	15	30	*115*
*Culex species*	Control	33	39	39	31	38	8	29	*217*
		327	102	119	88	96	87	127	*946*

### Insecticides resistance status

The deltamethrin and DDT had the lowest knockdown time (KT) in KT_50_, but higher in KT_90_ and KT_95_(Table 2). Permethrin (0.75%) and deltamethrin (0.05%) had 86.8 and 98.0% mortality after 24 h post exposure, respectively (Table 3).

**Table 2:** Knockdown time in minutes for *An. gambiae* s.l. wild population against different insecticides in susceptibility test using WHO kits

Insecticide	Knock down time in minutes (95% CI)			Goodness of fit test	
	KDT_50_	KDT_90_	KDT_95_	χ^2^	*P* value
Permethrin 0.75%	25.0 (17.0–31.9)	62.6 (50.5–83.4)	81.2 (64.0–117.6)	1101.8	<0.001
Deltamethrin 0.05%	23.7 (19.1–28.4)	63.5 (49.3–98.2)	83.9 (61.7–146.1)	1874.9	<0.001
DDT 4%	13.5 (8.92–17.77)	71.2 (66.11–79.21)	92.33 (85.44–99.39)	941.55	<0.011

**Table 3:** 24 h mortality for Wild population of *An. gambiae* s.l. after exposure to insecticides (mortality in other insecticides were 100% after 24 h)

Insecticide	Total mosquitoes tested	24 h mortality		95% CI	
		Number	%	Lower	Upper
Permethrin 0.75%	600	521	86.8	84.1	89.5
Deltamethrin 0.05%	600	588	98.0	96.9	99.1
DDT	600	600	100	–	–

### Mosquito density and biting cycles for 6 months in all three cases hourly

**Fig. 2:**
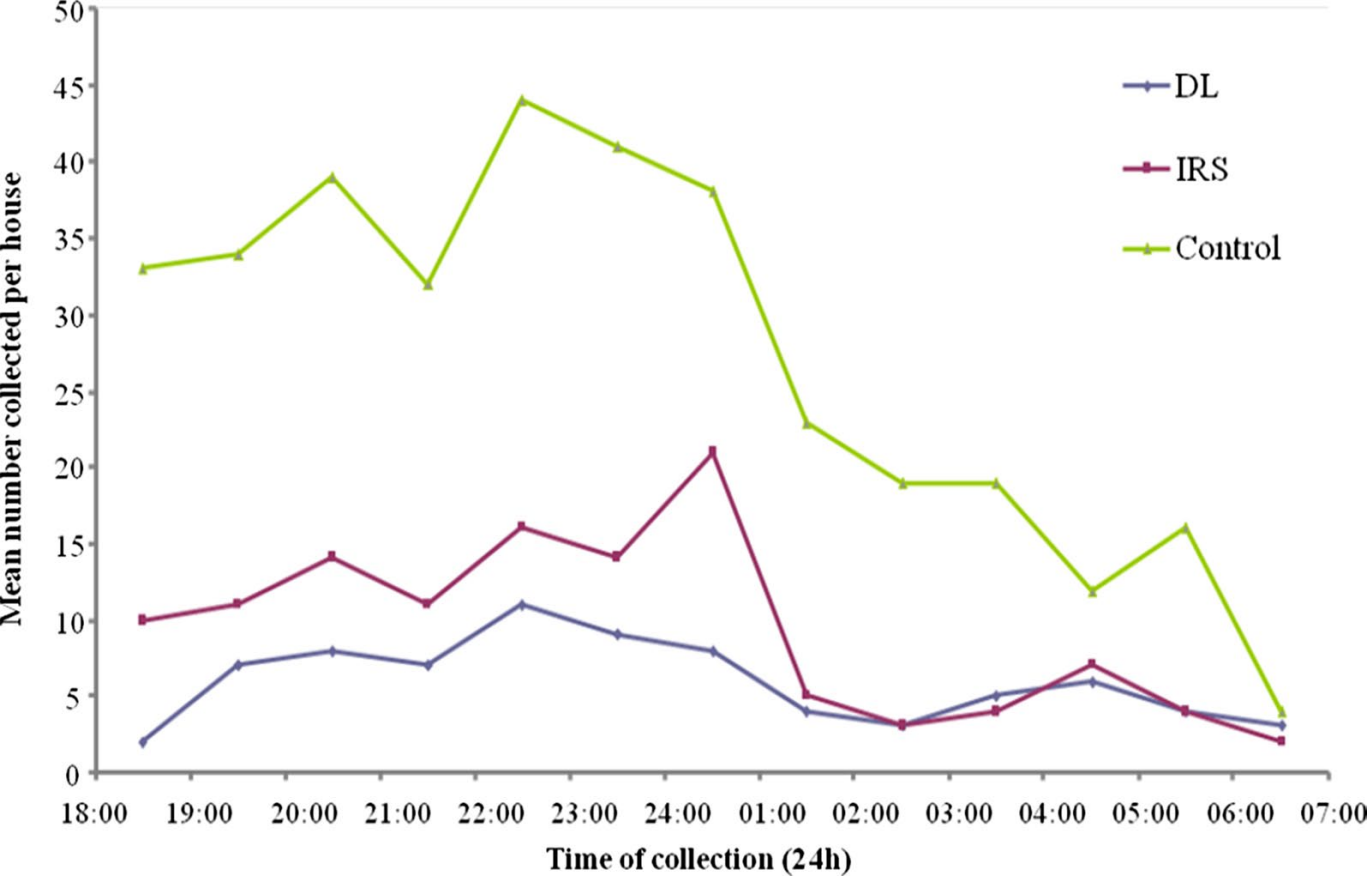
Graphs showing the hourly sampling of *An. gambiae* s.l. in houses treated with DL, IRS and control

**Fig. 3:**
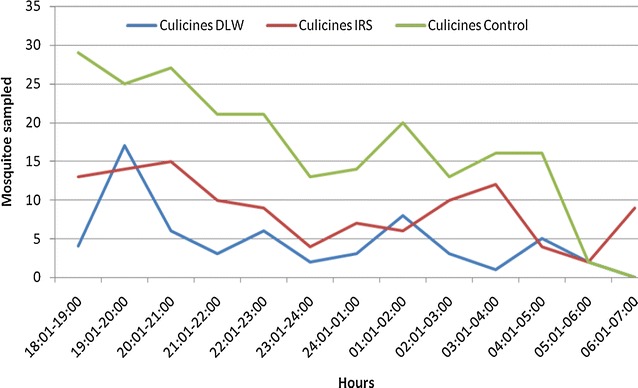
Graphs showing the hourly sampling of Culicine species in houses treated with DL, IRS and control

**Fig. 4:**
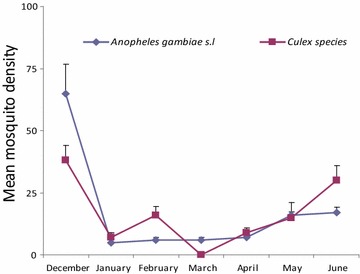
Graphs showing the differences between *An. gambiae* s.l. and culicine species in houses treated with IRS

**Fig. 5:**
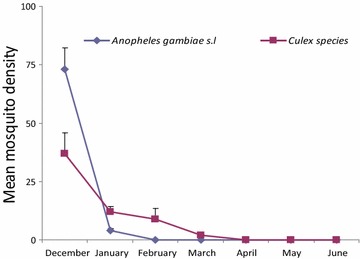
Graphs showing the differences between *An. gambiae* s.l. and culicine species dynamics in houses covered with durable wall lining

**Fig. 6:**
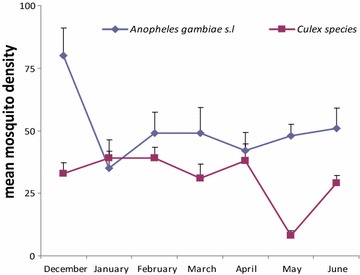
Graphs showing the differences between *An. gambiae* s.l. and culicine species dynamics in control houses

There was a difference in mosquito numbers in treatment houses soon after intervention conducted with IRS and DL compared to houses without treatment where the density remained higher but the biting frequency of *An. gambiae* s.l. and Culicine species still remained the same between the two arms (Figs. 2, 3). The mosquitoes biting frequency in hourly interval for *An.gambiae* s.l. was statistically significant higher in DL than IRS treatments (t = 2.649; df = 14, *P* = 0.021), the same trend was observed for *Culicine* species (t = 3.186, df = 14, *P* > 0.001). In the deltamethrin (IRS) sprayed houses, populations of both Culicines and *An. gambiae* s.l. were similar for the 6 month period of study (*P* = 0.819) (Fig. 4). Similar trend was observed in houses treated with DL (*P* = 0.214) (Fig. 5). In the control houses, the population of *An. gambiae* s.l. was slightly higher than that of Culicines, yet, the difference was statistically insignificant (*P* = 0.055) (Fig. 6).

### Contact bioassays

**Fig. 7:**
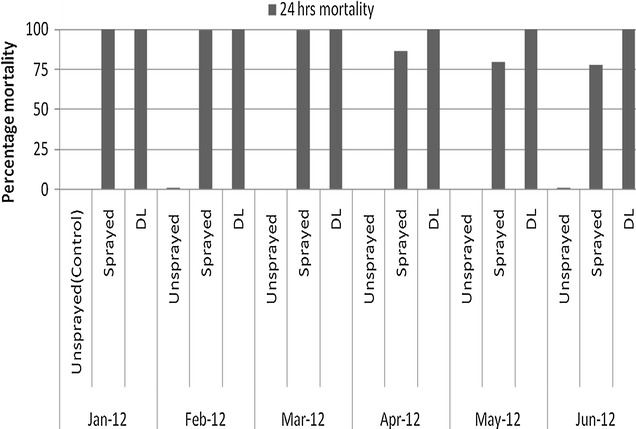
Twenty four hours (24 h) cone bioassay mortality of wild population of *An. gambiae* s.l. after exposure on sprayed walls and walls with DL

The exposed *An. gambiae* s.l. wild populations on the sprayed and DL surfaces mortality monthly varied significantly (Table 4). Mortality in sprayed surfaces was reduced as time elapsed while DL surfaces had no variation for the exposed mosquitoes for all 6 months (Fig. 7). For all 6 months the comparison between sprayed surface and DL mortalities were found to be significantly different (Fig. 7).

**Table 4:** Contact bioassays and mortality response for different surfaces with different treatments

Month	Surface		Number died	% Mortality	Lower 95% CI	Upper 95% CI	F	*P* value
Jan-12	Unsprayed (control)	800	0	0	0	0		
	Sprayed	800	800	100	0	0	151.18	<0.0001
	DL	800	800	100	0	0	151.18	<0.0001
Feb-12	Unsprayed (control)	800	6	0.75	0.55	0.97		
	Sprayed	800	796	99.5	99	100	195.03	<0.0001
	DL	800	800	100	0	0	197.02	<0.0001
Mar-12	Unsprayed (control)	800	0	0	0	0		
	Sprayed	800	798	99.75	99.21	100	199	<0.0001
	DL	800	800	100	0	0	200	<0.0001
Apr-12	Unsprayed (control)	800	0	0	0	0		
	Sprayed	800	693	86.63	74.33	98.82	152.83	<0.0001
	DL	800	800	100	0	0	200	<0.0001
May-12	Unsprayed (control)	800	3	0.38	0.24	0.5		
	Sprayed	800	638	79.75	68.65	90.82	131.17	<0.0001
	DL	800	800	100	0	0	198.49	<0.0001
Jun-12	Unsprayed (control)	800	8	1	0.61	1.39		
	Sprayed	800	624	78	71.2	87.2	124.05	<0.0001
	DL	800	799	99.88	99.8	100	195.56	<0.0001

## Discussion

The efficacy shown by DL material against wild population of *An. gambiae* s.l., is remarkably good for protection in wild population with low resistance frequency of deltamethrin [25, 26]. The durable wall lining (DL), has been found to have potential impact for mosquitoes control in one of malaria epidemic region of Tanzania. Mosquito densities caught indoor in a month pre interventions was higher than that caught during the six months of intervention. Following introduction of DL and IRS in the selected intervention houses, the decline in mosquito populations was remarkable. This trend was also observed in previous studies where interventions were implemented [2, 3, 13, 14, 27, 28]. Since both DL and IRS were pyrethroid treated, a deterrence effect for mosquitoes getting into these houses was possibly took place, thus contributing to the observed low indoors densities. The reduction in the indoor mosquitoes density indicates the efficacy of the two control tool (IRS and DL) in controlling mosquitoes. This has been revealed in other studies, which showed that, high pyrethroids intervention tools implementation reduced the proportions of indoor feeding mosquitoes [2, 3]. This can further be confirmed by the fact that, the hourly interval mosquitoes sampling did not show any significant differences in species abundance between *An. gambiae* s.l. and *Cx. quinquefasciatus* in treatment arms and control. In other study conducted parlay to this in Magugu by Mwanziva and other, revealed that 100% proportion of *An. gambiae* s.l. is composed of *An. arabiensis* [18]. Host seeking behaviour trend indoors did not change the peak time in treatment arms as they matched with control in spite of low density. This indicates that, intervention tools can reduce indoor resting mosquitoes population and did not changed the host seeking behaviour pattern of mosquitoes. This indicated that *An. gambiae* s.l. is naturally feed indoor but then deterred away after entering the house by indoor pyrethroids intervention to rest outdoor. This pattern of biting behavior has great implication on malaria transmission and it may somehow explain why we still have residual transmission going on despite the high coverage achieved. Similar observation has been reported in Ethiopia [9] and Sudan [17]. These findings are not contrary to what was found in Solomon Islands after DDT spray where *An. minimus* shifted from earlier hours host seeking in the morning to earlier hours of evening [29]. This is because that both DDT and pyrethroids insecticides possess strong deterrence efficacy.

Evaluation of resistance status of the wild population of *An. gambiae* s.l. in study area using WHO standard Kits, have revealed that, the population of *An. gambiae* s.l. have started showing indicators of resistance against permethrin, and deltamethrin which had highest knockdown time at KT_50_ (25, 23.7 and 8 min) and KT_95_ (83.9 81.2 and 10 min), respectively. DDT had higher knockdown time for 95% population to be knocked down (113.8 min) which shows to have threat of being tolerated by mosquitoes. This situation poses threat of cross-resistance and a single point mutation encoding the voltage-gated sodium channel which is now common mechanism of resistance in pyrethroids and DDT [30, 31]. After 24 h post exposure, the mortality was found to be 86.8 and 98% in permethrin and deltamethrin, respectively. These results indicated presence resistance to permethrin and an alert for deltamethrin. This situation needs further large scale study to justify resistance and susceptibility according to the updated WHOPES criteria for reporting of resistance and susceptibility data In spite of the new technology, intervention material which covers whole wall surface indoor, the threat of indoor malaria transmission might go on as resistance develops among mosquitoes species. The threat posed by deltamethrin and permethrin still of worrisome as these insecticides are widely used for IRS and LLINs for community mosquitoes control [32].

The epidemiological effect of the increased exophily in mosquito population due to pyrethroids indoor intervention has increased a transmission risk to outdoor and unprotected population. It has been observed in areas with high intervention coverage, mosquitoes are more exophagic than endophagic [2, 3, 33]. From this study, it shows that pyrethroids DL and IRS intervention tools once well used can lead to mosquitoes densities reduction indoors. This shall reduce indoor malaria transmissions. Nevertheless, according to Padonou and others [3], the scenario subsequently reduce disease transmission and increase risk to unprotected outdoors [33]. With this, personal repellents like N,N-Diethyl-3-methylbenzamide, menthol propyleneglycol carbonate can protect human from the risk of outdoor malaria transmission [34–36]. The use of mass trapping systems baited with human odours which attracts mosquitoes [37, 38] is of paramount importance in mosquitoes reduction outdoors. These traps might have value to mosquitoes control by incorporating bio pesticides in the systems such as fungi which have shown to cause high mortality in infected mosquitoes [39, 40]. The use of zoo prophylaxis have shown to have efficacy in areas with higher proportion of zoophilic mosquitoes [41]. The population reduction was attained after regular application of insecticides on cattle [42]. The number of animals kept outdoors has found significantly reducing *An. arabiensis* population indoors [43]. Therefore it’s important to consider combination of methods but with a right active ingredient and formulations in mosquitoes control as mosquitoes have greatly changed their traditional way of feeding and resting behaviour.

## Conclusion

In the light of these findings the selection of DL should be incorporated in integrated approach to malaria control for better protection. Further trials should be done in areas with higher mosquitoes population resistant to deltamethrin.

## Abbreviations

DL: durable lining
IRS: indoor residual spray
LLINs: long lasting insecticidal nets
WHOPES: WHO Pesticide Evaluation Scheme
DDT: dichloro diphenyl trichloroethane
KT: knockdown time
GLM: generalized linear model
